# Skin Pigmentation Differences between Mongolian, Korean, and Uzbekistan Ancient Human DNA Samples

**DOI:** 10.1155/2020/2585324

**Published:** 2020-08-11

**Authors:** Munkhtsetseg Bazarragchaa, Udval Uuganbayar, Kwang-Ho Lee, Kyung-Yong Kim, Kijeong Kim

**Affiliations:** ^1^Department of Molecular Biology and Genetics, School of Biomedicine, Mongolian National University of Medical Sciences, Ulaanbaatar, Mongolia; ^2^Department of Life Science, College of Natural Sciences, Chung-Ang University, Seoul, Republic of Korea; ^3^Institute of Gene and Genome Research, College of Medicine, Chung-Ang University, Seoul 06974, Republic of Korea

## Abstract

**Background:**

This study reports the use of real-time PCR to identify the SNP rs1545397 in the intron region on the OCA2 gene from ancient and degraded DNA isolated from ancient human bones from Mongolia, Korea, and Uzbekistan. This SNP is a marker for skin pigmentation. LightCycler-based probes (HybProbes) were designed. A LightCycler (version 2.0) system was used for the real-time PCR.

**Results:**

The results of the real-time PCRs of three different genotypes of SNP rs1545397 were compared with those of the direct sequencing. Melting curve analysis was used for genotype determination. Three genotypes were distinguished: the homozygous T (T/T) SNP type formed a distinct melting peak at 53.3 ± 0.14°C, the homozygous A (A/A) SNP type formed a distinct melting peak at 57.8 ± 0.12°C, and the heterozygous A/T SNP type formed two distinct melting peaks at 53.3 ± 0.17°C and 57.8 ± 0.15°C. Mongolian aDNA samples tested in this study carried all three types of the SNP (A/T, A/A, and T/T) with no distinctly predominant type observed. In contrast, Korean aDNA samples carried the Asian genotype (T/T), while the Uzbekistan aDNA samples carried the European genotype (A/A) more often than the Asian genotype (T/T).

**Conclusions:**

Human Mongolian aDNA samples had A/T, A/A, and T/T SNP rs1545397 with no distinct predominant genotype. When combined with the archeological and aDNA studies of other coupling morphologies with aDNA, our results infer that Mongolia's prehistoric population had considerable heterogeneity of skin color and morphological traits and that in the Neolithic period, a Eurasian or mixed population inhabited the western part of Mongolia.

## 1. Introduction

Ancient DNA samples have provided information about human migration and genetic relationship between modern *Homo sapiens* and their closest extinct relatives that inhabited Eurasia, the Neanderthals, and their hominid cousins, the Denisovans [1, 2]. Since DNA gathers mutations over time, which is then inherited, it contains historical information. By comparing DNA sequences, geneticists can infer the evolutionary history of an organism, their phylogeny.

Archeologists have found many artifacts related to prehistoric times throughout Mongolia. The prehistoric population of Mongolia from the Neolithic period (8000-6000 BC) to the Xiongnu period (200 AD) revealed notable heterogeneity of morphological traits [3, 4]. Archeological studies show that in the Bronze and Early Iron Age cultures, there was a significant cultural difference between the western and eastern parts of Mongolia [5, 6]. Western Mongolia Bronze and Early Iron Age cultures belong to the Altai-Sayan variant of the South Siberian Bronze and Iron Age cultures. This culture is associated with the stone kurgans, deer stone monument, and rock art, which they widely distributed. In Eastern and Central Mongolia, there was a so-called Slab Grave culture. This culture not only was widely distributed all over Eastern and Central Mongolia but also covered the Lake Baikal region in the north, the Ordos in the south, the Khangai Mountain region in the west, and Manchuria in the east.

Ancient DNA is a powerful tool for studying the history of how people moved around and interacted in the ancient world. Most ancient DNA (aDNA) studies used single nucleotide polymorphism (SNP) analysis and short tandem repeat (STR) profiles of mitochondria, autosomes, and Y chromosomes. These methods are widely used for studies of human evolution, migration, and population histories and forensic sciences.

Subsequently, various potentially informative SNPs on several human pigmentation candidate genes were studied. These studies show striking polymorphism in large allele frequencies between the populations [7, 8]. The distribution of skin pigmentation differs from other phenotypic traits and most other genetic markers. About 88% of the total variation in skin pigmentation can be explained by pigmentation differences among major geographic groups [9]. Pigmentation is a polygenic trait, but the number of genes and the exact nature of the allelic variants determining melanin content remain, for the most part, unknown.

However, some genetic determinants of skin pigmentation are known. One single nucleotide polymorphism haplotype in an intron of the oculocutaneous albinism (OCA2) gene on chromosome 15 of *Homo sapiens* has been identified. The OCA2 gene (formerly called the P gene) encodes the P protein located in melanocytes, which are specialized cells that produce a pigment called melanin. Melanin is the substance that gives skin, hair, and eyes their color. A fragment that includes SNP rs1545397 (c.1951+9158T>A) located in the intron region on the OCA2 gene has been studied. Mutation in this gene results in type 2 oculocutaneous albinism [7–11].

The current evidence indicates that OCA2 is highly polymorphic and a key determinant of the variation in eye color and also plays a role in skin pigmentation variation (6). The SNP rs1545397 showed large allele frequency differences between Asians, which have high prevalence of TT alleles, and modern Europeans and others, with high prevalence of A/A alleles [12].

Our study is aimed at using real-time PCR on ancient degraded human DNA excavated from Mongolia, Korea, and Uzbekistan to determine the SNP rs1545397, in the intron region on the OCA2 gene, to infer skin pigmentation differences in those ancient populations.

## 2. Materials and Methods

### 2.1. Ancient Human Samples and Sample Preparation

A total of 58 ancient human DNA (aDNA) samples used in this study, excavated from Mongolia, Korea, and Uzbekistan, were analyzed in this study (Table S1 supplementary material).

### 2.2. Contamination Control

The bone samples were collected with extensive precautions according to previously published procedures to prevent contamination of DNA samples [13, 14].

The bone's dirty surface was entirely removed using burrs to remove humic acid substances, which are dark-colored breakdown products of decaying organic matter that is the primary cause for inhibition of DNA extraction from soil or buried biological remains. Other contaminants were removed from bone fragments by immersion in undiluted bleach for at least 30 min and then rinsing the fragments with sterile distilled water. The bones were then air-dried under UV irradiation at 254 nm for 1 hour within a closed sterile cabinet and then were stored in the sterile cabinet without UV light overnight. The samples were placed into sterile grinding jars and then were immersed in liquid nitrogen for 5 minutes and were ground into a fine powder using the Mixer Mill MM301 (Retsch). Before grinding the bone into powder, the mixer mill jars and balls were autoclaved for 2 hours to protect from endogenous sources.

The aDNA from the bone samples were collected and amplified by real-time PCR with extensive precautions in two separate laboratories to prevent contamination. This was done in cleanrooms for pre- and post-PCR experiments, which were specifically dedicated to aDNA experiments only. The cleanrooms were equipped with an air filtration system. All researchers handling the materials or working in the laboratory wore protective clothing, including UV-irradiated lab gowns, face and mouth masks, and latex gloves. Workplaces, containers, and appliances were cleaned by using an undiluted commercial bleach and UV-irradiated at 254 nm for one hour. All steps (bone cutting, surface removing, powdering, DNA extraction, PCR preparation, and post-PCR works) were carried out in a laminar airflow clean bench in a separate clean room. Throughout all manipulations, sterile aerosol barrier (filter tip) pipets and pipet tips were used. Reagent blanks (negative controls) containing all the steps except bone powder were included throughout the process to monitor potential material or worker-originated human DNA contamination.

### 2.3. DNA Extraction from Ancient Human Samples

The bone powders were incubated with extraction buffer QG (Qiagen Co., Valencia, CA, USA). After incubating, the supernatant was added to a binding solution, and 90 *μ*l silica suspension was added. This mixture was incubated for 3 hours at room temperature under rotation. After centrifugation, the supernatant was discarded. The silica pellet was washed with 0.7 ml binding solution. After removing the binding solution, the pellet was resuspended in wash solution (PE buffer), and the supernatant was again removed. Subsequently, the silica pellet was air-dried at room temperature for 30 minutes on the clean bench. The completely dried silica pellet was completely resuspended in 120 *μ*l EB buffer and incubated for 5 minutes at room temperature. The supernatant was collected without any silica particles. The pellet was again suspended in the EB buffer, and the supernatant was again collected. We then used the QIAquick PCR Purification Kit.

### 2.4. Oligonucleotide Probe Design

The PCR products produced from 58 aDNA samples were analyzed for their SNP genotype by probe-based real-time PCR. LightCycler-based probes (HybProbes) were designed for the PCR. When designing the HybProbes, we ensured that the anchor and sensor probes were adjacently hybridized to the complementary target DNA for strong fluorescence emission by a mechanism of fluorescence resonance energy transfer (FRET). The potential presence of cross-complementarities among all the primers and probes was checked by using LC PDS (version 2.0) software. The sequence of probes was modified while maintaining the probe specificities to obtain high sensitivity. The differential melting temperatures (Tm) of the designed probes for the SNP determination were checked by calculation before the real-time PCR experiment (Table 1).

### 2.5. Real-Time Polymerase Chain Reaction of Three Different Genotypes at the rs1545397 Region

A LightCycler (version 2.0) system was used for real-time PCR, and its four detection channels were calibrated for color compensation and activated for the experiment. The LightCycler FastStart DNA Master HP kit (Roche Diagnostics) was used to prepare the master mixture, according to the protocol provided with the kit. A 10 *μ*l reaction mixture was prepared for each sample containing the following: 1 *μ*l Taq buffer (which contained a deoxynucleoside triphosphate mixture and 10 mM MgCI_2_), an additional 2 mM MgCI_2_, 0.75 *μ*M forward primer, 0.35 *μ*M reverse primer, 0.2 *μ*M anchor probe and sensor probe, tenfold diluted PCR product as a template, and sterile distilled water. The cycling conditions were 10 minutes at 95°C, and 35 cycles of 10 s at 95°C, 10 s at 62°C, and 12 s at 72°C. A melting curve analysis followed by cycling for 10 s at 95°C and 30 s at 45°C was performed. The temperature was then increased from 45°C to 90°C at a temperature transition rate of 0.10°C/s, during which the fluorescence signal was continuously acquired. The real-time PCRs of three different genotypes at rs1545397 were compared with those of the direct sequencing.

### 2.6. Evaluation of the PCR Performance

Our previous reports described the choice of DNA polymerases for highly degraded human samples. We used Ex Taq HS, FastStart, PicoMaxx HF, and AmpliTaq Gold DNA polymerase with 58 positive samples tested in duplicate four times [15].

For the PCR results, the degree of PCR inhibition was evaluated based on the quantification cycle (Cq) and end cycle fluorescence (ECFL). The Cq was used for the quantification of the template DNA in quantitative PCR (qPCR). The Cq increased as the template quantity decreased or PCR inhibition occurred. The ECFL was defined as the fluorescence at the final cycle of amplification, and it reflected the amount of the final PCR product. The ECFL decreased as PCR inhibition occurred [16].

## 3. Results and Discussion

One of our investigators Kijeong K, previously developed a silica-based method of DNA extraction using ion-exchange columns; however, it required extended extraction time and was laborious [14]. Instead, we developed a straightforward and efficient method to extract DNA recovery from old bone samples, and we used the QIAquick PCR Purification Kit instead of ion-exchange columns. This method was simpler and more efficient.

**Figure 1:**
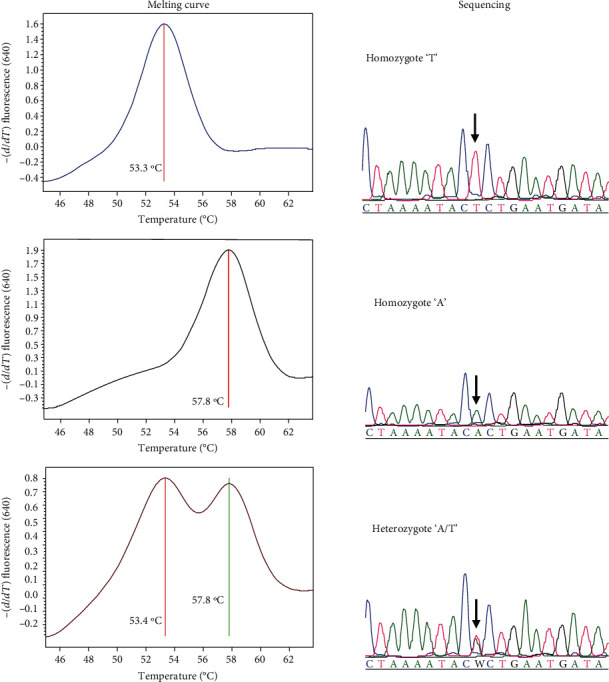
Real-time PCR melting curve analysis to identify the SNP rs1545397 and its confirmation by DNA sequencing.

The application of the real-time PCR method designed to detect the three types of SNP rs1545397 demonstrated a clear separation among the three SNP types, showing distinct melting temperatures according to the degree of probe hybridization. The homozygous “T” (T/T) type formed a distinct melting peak at 53.3 ± 0.14°C at Ch. 640. The homozygous “A” (A/A) SNP type formed a distinct melting peak at 57.8 ± 0.12°C at Ch. 640. The heterozygous “A/T” SNP type formed two distinct melting peaks at 53.3 ± 0.17°C and 57.8 ± 0.15°C at Ch. 640 (Figure 1).

**Figure 2:**
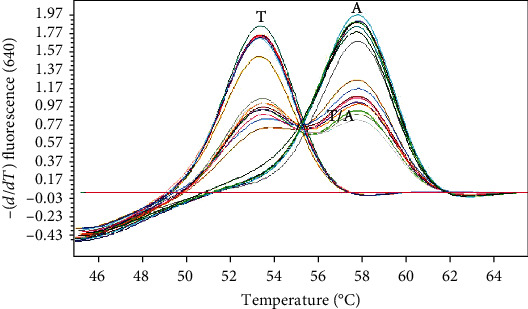
Representative melting curve analysis of aDNA samples for the determination of SNP rs1545397.

The aDNA melting curve analysis results for the real-time PCR matched the results of the sequencing analysis entirely (Figure 2).

The genotype data of the 58 ancient samples are found in (Table 2). The homozygous T/T and A/A or the heterozygous A/T genotypes for rs1545397 SNP were identified.

We analyzed 58 ancient human DNA samples, of which 28 were from Mongolia, 8 from Korea, and 22 from Uzbekistan. Among the 28 Mongolian aDNA samples, 10 were A/A (European) alleles, 14 were T/T (Asian) alleles, and 4 were A/T (heterozygote). The 8 Korean aDNA samples all carried the Asian genotype (T/T). Among the 22 Uzbekistan samples, 11 were A/A (European) alleles, 2 were T/T (Asian) alleles, and 9 were A/T (heterozygote), respectively (Table 3).

Many researchers have studied prehistoric human remains belonging to different historical periods of Mongolia. The paleoanthropological studies of human remains of Mongolia from the Neolithic, Bronze, and Early Iron Ages reveal great heterogeneity of morphological traits among the population of these historical periods. This heterogeneity is known because cranial studies, used to provide a comparative foundation for Mongolian prehistoric remains, encompass craniofacial data on prehistoric populations from Central Asia, South Siberia, Russian Far East, China, Korea, and Japan [3].

In the Neolithic period, people with paler skin with European morphological features inhabited Western Mongolia, while the population with more developed Mongol traits occupied Central and Eastern Mongolia. In the Bronze Age, Western Mongolia's population exhibited more pronounced Mongolian morphological features than earlier times. As a result of our study, we agree with the hypothesis of Mongolian archeologists that the early Bronze Age was characterized by movements from Eastern Mongolia to Western Mongolia, where intensive mixing between local European and Asian ancestry populations took place [3, 4]. The admixture probably continued until the end of the Xiongnu period. The Xiongnu population of Mongolia anthropologically and ethnically was heterogeneous because their empire ruled over a vast territory, including diverse nomadic tribes, for 300 years.

The Xiongnu period is one of the most investigated historical periods of Mongolia. The Xiongnu played an important role in the ethnic and political history and culture of Eurasia. Xiongnu migration from Mongolia to the West through Altai and Tuva played an important role in the ethnogenetical process and the anthropological structure of the region [4].

In 1943, a Mongolian Russian expedition discovered a Xiongnu cemetery at Egyin Gol necropolis, in Northern Mongolia, and mtDNA analysis shows a majority (89%) of the Xiongnu population belonged to an Asian haplogroup (A, B4b, C, D4, D5 or D5a, and F1b), and 11% belonged to European haplogroups (U2, U5a1a, and J1). Researchers concluded that these results indicate that the contact between European and Asian populations occurred before the Xiongnu culture [17, 18].

In 1974, Mongolian archeologists discovered a 2000-year-old Xiongnu elite cemetery at Duurleg Nars in Bayan-Adarga sum north of Khenti Aimag. The DNA analyses revealed that one subject was an ancient male skeleton with maternal U2e1 and paternal R1a1 haplogroups. R1a is the most common haplogroup in Europe. The author concluded that this finding was indicative of the migration of Indo-European people. Other specimens were a female with mtDNA haplogroup D4 and a male with Y-SNP haplogroup C3 and mtDNA haplogroup D4. Those haplogroups were common in Northeast Asia and no close kinship among them [13, 19].

In 2004, the Mongolian excavation team from the Department of Anthropology and Archaeology, National University of Mongolia, discovered five graves belonging to the Mongol imperial family (designated the Golden family) in Tavan Tolgoi, Eastern Mongolia. Researchers defined the genealogy of the five bodies and their kinship using SNP and STR profiles of mitochondria, autosomes, and Y chromosomes. The result showed East Asian D4 or CZ matrilineal and West Eurasian R1b-M343 patrilineal origins. It revealed a genealogical mixture of Caucasoid and Mongoloid ethnic groups [20].

The mixing of Asian and European continental ancestry groups may be inherent in the genetic structure of modern-day Mongolians. According to the few molecular studies of modern Mongolian populations, Mongolian populations carried their distinctive haplogroups worldwide [17, 18, 21].

Our first and successful report of pigment phenotype analysis of ancient samples using probe-based real-time PCR instead of DNA sequencing analysis showed that most modern Europeans carried the A/A homozygous allele. In contrast, Asians carried the T/T homozygous allele in rs1545397 in the OCA2 gene.

Interestingly, Mongolian aDNA samples tested in our study carried all three types of the SNP (A/T, A/A, and T/T) with no distinct predominant type observed. In contrast, aDNA samples from Koreans carried the Asian genotype (T/T), while the European (AA) genotype was found in the human aDNA from Uzbekistan rather than the Asian genotype (T/T).

In future studies, various SNPs in pigmentation multiple candidate genes from aDNA samples can be identified simultaneously, conveniently, rapidly, sensitively, and specifically if a probe-based real-time PCR like the FRET-based real-time PCR is used.

## 4. Conclusions

Human Mongolian aDNA samples had A/T, A/A, and T/T SNP rs1545397 with no distinct predominant genotype. When combined with the archeological and aDNA studies of other coupling morphologies with aDNA, our results infer that Mongolia's prehistoric population had considerable heterogeneity of skin color and morphological traits and that in the Neolithic period, a Eurasian or mixed population inhabited the western part of Mongolia.

## Figures and Tables

**Table 1:** Primers and HybProbes used for the amplification and detection of rs1545397.

Primers/Probes	Sequence (5′-3′)	Tm (°C)^a^	Note
Primers			
rs1545397_F	TGGAATTGGATACTGACAATGGTTG	64.4	Bouakaze et al. 2009
rs1545397_R	CATGGGGGAGAGAGAATGACTCAG	67.7	Bouakaze et al. 2009
Probes			
rs1545397_A	ATTCACACAACATAAAATTTATCTTGCAA-FL^b^	60	This study complementary sequence
rs1545397_S	LC Red640^c^-ATTATATCATTCAGTGTATTTTAGTATATT-PH^d^	55(T) 50.5(A)	Channel 640

a: calculated by using LC PDS software (version 2.0). b: FL, fluorescein. c: LC Red640, LightCycler dye Red640. d: PH, phosphate.

**Table 2:** SNP rs1545397 of DNA samples determined by real-time PCR.

No.	Sample code	Tm	SNP	No.	Sample code	Tm	SNP	No.	Sample code	Tm	SNP
1	KR153	53.31	T/T	21	MN322	57.86	A/A	41	UZ189	57.87	A/A
2	KR69	53.39	T/T	22	MN324	57.88	A/A	42	UZ294	57.82	A/A
3	MN298	57.75	A/A	23	MN262	53.43	T/T	43	UZ57	54.4/57.5	A/T
4	MN323	57.86	A/A	24	MN338	53.43	T/T	44	UZ111	57.83	A/A
5	MN313	53.49	T/T	25	MN345	57.7	A/A	45	UZ177	53.4/57.9	A/T
6	KR64	53.34	T/T	26	MN250	53.35	T/T	46	UZ304	53.4/57.8	A/T
7	KR39	53.34	T/T	27	MN377	53.38	T/T	47	UZ282	57.75	A/A
8	KR21	53.25	T/T	28	MN373	57.67	A/A	48	UZ232	57.8	A/A
9	KR69	53.34	T/T	29	MN378	57.79	A/A	49	UZ376	57.91	A/A
10	MN143	57.64	A/A	30	MN226	53.42	T/T	50	UZ378	53.5/57.8	A/T
11	MN131	53.31	T/T	31	MN351	53.5/57.8	A/T	51	UZ379	53.5/57.8	A/T
12	MN139	53.25	T/T	32	MN369	53.35	T/T	52	UZ384	57.9	A/A
13	MN160	53.4/57.7	A/T	33	MN312	53.36	T/T	53	UZ105	57.8	A/A
14	UZ221	53.5/57.8	A/T	34	MN329	53.34	T/T	54	UZ106	57.6	A/A
15	UZ223	53.4/57.9	A/T	35	MN343	57.76	A/A	55	UZ107	53.5/57.9	A/T
16	UZ187	53.43	T/T	36	MN344	53.5/57.8	A/T	56	KR008	53.5	T/T
17	UZ301	53.4/57.8	A/T	37	MN253	53.42	T/T	57	UZ232	57.71	A/A
18	UZ286	53.45	T/T	38	MN255	57.72	A/A	58	KR189	53.4	T/T
19	MN317	53.33	T/T	39	MN257	T/T					
20	MN319	53.4/57.8	A/T	40	UZ193	A/A					

**Table 3:** Distribution of SNP of rs1545397 in aDNA samples.

	A/A (European)	T/T (Asian)	A/T
Mongolian aDNA	10 (36.0%)	14 (50.0%)	4 (14.0%)
Korean aDNA	0	8 (100%)	0
Uzbekistan aDNA	11 (50.0%)	2 (9.0%)	9 (41.0%)

## Data Availability

The data used in this study are provided in the figures, tables, and supplementary materials.

## Supplementary Materials

Supplementary Materials Table S1: list of ancient human DNA samples excavated from Mongolia, Korea, and Uzbekistan used in this study. It contains 58 ancient bone samples, of which 28 are from Mongolia, 8 from Korea, and 22 from Uzbekistan, and information about their excavation sites, ages, and bone fragments. Table S2: results of real-time PCR analysis of positive samples with four polymerases.

## References

[1] Prüfer K., Racimo F., Patterson N. (2014). *The complete genome sequence of a Neanderthal from the Altai Mountains*. *Nature*.

[2] Meyer M., Kircher M., Gansauge M. T. (2012). *A high*-*coverage genome sequence from an archaic Denisovan individual*. *Science*.

[3] Tumen D. (2006). *Paleoanthropology of the ancient population of Mongolia*. *Mongolian Journal of Anthropology, Archaeology and Ethnology*.

[4] Tumen D., Khatanbaatar D., Erdene M., Ankhsanaa G., Vanchigdash C. (2011). *Ancient Bronze Age and Xiongnu cultures in Mongolia*.

[5] Volkov V. V. (1981). *Olennie Kamni Mongolii [Deer Stones of Mongolia]*.

[6] Erdenebaatar D. (2002). *The Four Sided Grave and Khirigsuur Cultures of Mongolia*.

[7] Parra E. J. (2007). *Human pigmentation variation*: *evolution*, *genetic basis*, *and implications for public health*. *American Journal of Physical Anthropology*.

[8] Bouakaze C., Keyser C., Crubézy E., Montagnon D., Ludes B. (2009). *Pigment phenotype and biogeographical ancestry from ancient skeletal remains*: *inferences from multiplexed autosomal SNP analysis*. *International Journal of Legal Medicine*.

[9] Edwards M., Bigham A., Tan J. (2010). *Association of the OCA2 polymorphism His615Arg with melanin content in east Asian populations*: *further evidence of convergent evolution of skin pigmentation*. *PLoS Genetics*.

[10] Duffy D. L., Montgomery G. W., Chen W. (2007). *A three*-*single*-*nucleotide polymorphism haplotype in intron 1 of OCA2 explains most human eye*-*color variation*. *American Journal of Human Genetics*.

[11] Lee S. T., Nicholls R. D., Jong M. T. C., Fukai K., Spritz R. A. (1995). *Organization and sequence of the human P gene and identification of a new family of transport proteins*. *Genomics*.

[12] Yuasa I., Umetsu K., Harihara S. (2007). *Distribution of two Asian*-*related coding SNPs in the MC1R and OCA2 genes*. *Biochemical Genetics*.

[13] Kim K., Brenner C. H., Mair V. H. (2010). *A western Eurasian male is found in 2000*-*year*-*old elite Xiongnu cemetery in Northeast Mongolia*. *American Journal of Physical Anthropology*.

[14] Kim K., Kim K. Y., Jeon E. (2008). *Technical note*: *improved ancient DNA purification for PCR using ion*-*exchange columns*. *American Journal of Physical Anthropology*.

[15] Kim K., Bazarragchaa M., Brenner C. H., Choi B. S., Kim K. Y. (2015). *Extensive evaluation of DNA polymerase performance for highly degraded human DNA samples*. *Forensic Science International*.

[16] Kim K. Y., Kwon Y., Bazarragchaa M. (2013). *A real*-*time PCR*-*based amelogenin Y allele dropout assessment model in gender typing of degraded DNA samples*. *International Journal of Legal Medicine*.

[17] Keyser-Tracqui C., Crubezy E., Ludes B. (2003). *Nuclear and mitochondrial DNA analysis of a 2*,*000*-*year*-*old necropolis in the Egyin Gol Valley of Mongolia*. *American Journal of Human Genetics*.

[18] Kolman C. J., Sambuughin N., Bermingham E. (1996). *Mitochondrial DNA analysis of Mongolian populations and implications for the origin of New World founders*. *Genetics*.

[19] Bazarragchaa M., Kim K., Kim J. H. (2009). *A kinship analysis of ancient human bones and teeth from Mongolia*. *Korean J Phys Anthropol*.

[20] Lkhagvasuren G., Shin H., Lee S. E. (2016). *Molecular genealogy of a Mongol queen's family and her possible kinship with Genghis Khan*. *PLoS One*.

[21] Keyser-Tracqui C., Crubézy E., Pamzsav H., Varga T., Ludes B. (2006). *Population origins in Mongolia*: *genetic structure analysis of ancient and modern DNA*. *American Journal of Physical Anthropology*.

