# Supplementary material for: Skin Pigmentation Differences between Mongolian, Korean, and Uzbekistan Ancient Human DNA Samples
Source: Biomed Res Int. 2020 Aug 11;2020:2585324. doi: 10.1155/2020/2585324 (PMC7559177; doi:10.1155/2020/2585324)
Supplement: Supplementary Materials — Table S1: list of ancient human DNA samples excavated from Mongolia, Korea, and Uzbekistan used in this study. It contains 58 ancient bone samples, of which 28 are from Mongolia, 8 from Korea, and 22 from Uzbekistan, and information about their excavation sites, ages, and bone fragments. Table S2: results of real-time PCR analysis of positive samples with four polymerases. [file 2585324.f1.pdf]

Table S1. List of ancient human DNA samples excavated from Mongolia, Korea, and Uzbekistan

№	Code	Excavation site	Age	Bone
1	KR153	Singal, Korea	15c-19c, Chosun	Lt.tibia
2	KR69	Donghae, Korea	6c, Shilla	Rt.femur
3	MN298	Uvs, Chandmani, Mongolia	Late bronze-early iron	Femur
4	MN323	Arkhangai, Ogiinuur, Mongolia	Xiongnu	Femur
5	MN313	Ovorkhangai, Kharakhorum, Mongolia	Mongol	Frontal bone
6	KR64	Donghae, Korea	6c, Shilla	Rt.femur
7	KR39	Kongju, Korea	5c, Baekjae	Rt. ulnar
8	KR21	Sachun, Korea	2c, early iron	Rt. humerus
9	KR69	Donghae, Korea	6c, Shilla	Rt.femur
10	MN143	Ovorkhangai, Bogd, Chandmani, Mongolia	Xiongnu	Rt. tibia
11	MN131	Sukhbaatar Tavan tolgoi, Mongolia	Mongol	Lt. tibia
12	MN139	Bayankhongor Galuut, Mongolia	Xiongnu	Lt. tibia
13	MN160	Uvs, Ulaangom, Chandmani, Mongolia	Late bronze- early iron, Mongolia	Rt. tibia
14	UZ221	ChorBakir, Buhara	16-17c	Molar teeth
15	UZ223	ChorBakir, Buhara,	16-17c	Molar teeth
16	UZ187	Kulsuyuksoi, Djizak, Uzbekistan	modern	Molar teeth
17	UZ301	Karakalpakistan, north Uzbekistan	Bronze	Molar teeth

№	Code	Excavation site	Age	Bone
18	UZ286	Shayhantauk, Tashkent Karatepa, old	17-18c	Molar teeth
19	MN317	Hentii, Jargaltkhaan, Mongolia	Xiongnu	Radius
20	MN319	Hentii, Delgerkhaan, Mongolia	Mongol	Femur
21	MN322	Arkhangai, Battsengel, Mongolia	Xiongnu	Femur
22	MN324	Arkhangai, Battsengel, Mongolia	Xiongnu	Femur
23	MN262	Dornogobi, Erdenedalai, Hadan Usnii hooloi, Mongolia	Mongol	Femur
24	MN338	Dornod kholonbuir, Santiin am, Mongolia	Xiongnu	Radius
25	MN345	Dornod kholonbuir, Uguumur, Mongolia	Mongol	Metacarpal
26	MN250	Bulgan, Saikhan, Haltar, Mongolia	Xiongnu	Femur
27	MN377	Selenge, Uguumur, Sant Mongolia	Xiongnu	Femur
28	MN373	Arkhangai Tamriin ulaan khoshuu, Mongolia	Xiongnu	Femur
29	MN378	Selenge, Uguumur, Sant Mongolia	Xiongnu	Femur
30	MN226	Ovorkhangai, Kharakhorium, Mongolia	Mongol	Femur
31	MN351	Bayan-Olgii, Khurgak gobi, Mongolia	Bronze	Humerus
32	MN369	Sukhbaatar, Asgat,Sharga mountain, Mongolia	Mongol	Femur
33	MN312	Ovorkhangai, Kharakhorum, Mongolia	Mongol	Femur

№	Code	Excavation site	Age	Bone
34	MN329	Dornod, kholonbuir, Tsuvraa uul, Mongolia	Mongol	Femur
35	MN343	Arkhangai, Battsengel, Solibo uul, yamaatiin am, Mongolia	Xiongnu	Femur
36	MN344	Arkhangai, Battsengel, Solibo uul, yamaatiin am, Mongolia	Xiongnu	Femur
37	MN253	Khovsgol, Renchinlkhumbe, Mongolia	Modern	Femur
38	MN255	Arkhangai, Ondorkhaan, Hanui brigad, Mongolia	Mongol	Clavicle
39	MN257	Khowd, Mankhan, Tahiltiin hotgor, Mongolia	Mongol	Femur
40	UZ193	Shoxruhiya, Uzbekistan	16-17c	Molar teeth
41	UZ189	Kaltepa, Djizak, Uzbekistan	Modern	Molar teeth
42	UZ294	Termez, Surhandarya region, south Uzbekistan	15-17c	Molar teeth
43	UZ57	Sarazm, Uzbekistan	Bronze	Molar teeth
44	UZ111	Djarkutan, Uzbekistan	15-16c	Molar teeth
45	UZ177	Kulagaytepa, Napay region, Samarkand, Uzbekistan	15-16c	Molar teeth
46	UZ304	Namangan, Uzbekistan	15-16c	Molar teeth
47	UZ282	Paltaz, Uzbekistan	5-6 c	Molar teeth
48	UZ232	Dashti-Kazi Pendjikent, Tadjikstan	bronze	Molar teeth

№	Code	Excavation site	Age	Bone
49	UZ376	Old Termez, Surhandary, south Uzbekistan	9-10c	Molar teeth
50	UZ378	Djarkuntan 4, Surhandarya, south Uzbekistan	Bronze	Molar teeth
51	UZ379	Djarkuntan 4, Surhandarya, south Uzbekistan	Bronze	Molar teeth
52	UZ384	Parhai, south Turkmenistan Djarkutan 4, Surhandarya, south Uzbekistan	Bronze	Molar teeth
53	UZ105	Djarkutan, Uzbekistan	15-16c	Molar teeth
54	UZ106	(H.G) Povoljie, Uzbekistan	15-16c	Molar teeth
55	UZ107	(H.G) Povoljie, Uzbekistan	15-16c	Molar teeth
56	KR008	Sachun, Korea	2c, early iron	Lt. tibia
57	UZ232	Dashti-Kazi Pendjikent, Tadjikstan	Bronze	Molar teeth
58	KR189	Najoo, Korea	Baekje	Lt. humerus

3 **MN Mongolian, KR Korean, UZ Uzbekistan**

Table S2. Result of real-time PCR analysis of positive samples with four polymerases

			Ampli*Taq* Gold		FastStart *Taq*		Ex *Taq* HS		Pico Maxx HF	
**№**	**Sample**	**T_m(^OC)**	**Cp**	**ECFL**	**Cp**	**ECFL**	**Cp**	**ECFL**	**Cp**	**ECFL**
**1.**	**KR153**	53.31	35.9	0.32	36.4	0.29	33.8	0.43	35.8	0.15
**2.**	**KR69**	53.39	34.4	0.77	35.2	0.92	34.0	0.88	34.0	0.46
**3.**	**MN298**	57.75	33.2	0.76	34.5	1.10	34.1	1.20	34.2	0.98
**4.**	**MN323**	57.86	30.1	0.67	29.7	0.85	29.7	0.77	29.7	0.75
**5.**	**MN313**	53.49	28.8	0.87	29.1	1.10	28.7	0.93	28.8	0.72
**6.**	**KR64**	53.34	34.0	0.70	35.0	0.90	34.0	0.80	34.2	0.51
**7.**	**KR39**	53.34	34.0	0.72	35.5	0.94	34.1	0.82	34.0	0.53
**8.**	**KR21**	53.25	33.5	0.22	35.8	0.41	35.0	0.62	34.8	0.54
**9.**	**KR69**	53.34	34.4	0.77	35.2	0.92	34.0	0.88	34.0	0.46
**10.**	**MN143**	57.64	33.2	0.25	32.8	0.51	31.9	0.74	32.6	0.73
**11.**	**MN131**	53.31	31.1	0.18	31.1	0.53	30.5	0.83	31.4	0.71
**12.**	**MN139**	53.25	35.4	0.53	34.7	0.93	35.0	0.61	34.01	0.57
**13.**	**MN160**	53.4/57.7	34.1	0.35	33.8	0.60	34.9	0.86	33.8	0.88
**14.**	**UZ221**	53.5/57.8	31.8	0.16	31.9	0.27	32.1	0.67	32.7	0.46
**15.**	**UZ223**	53.4/57.9	32.3	0.18	34.6	0.53	36.7	0.63	34.2	0.62
**16.**	**UZ187**	53.43	29.6	0.08	31.4	0.49	30.1	0.65	29.8	0.58
**17.**	**UZ301**	53.4/57.8	29.9	0.40	30.7	0.56	30.3	0.89	30.3	0.79
**18.**	**UZ286**	53.45	32.9	0.30	34.5	0.51	33.2	0.74	34.0	0.64
**19.**	**MN317**	53.33	36.0	0.59	30.0	0.52	30.9	0.86	31.2	0.74
**20.**	**MN319**	53.4/57.8	31.8	0.51	32.3	0.69	32.2	0.97	32.6	0.81
**21.**	**MN322**	57.86	33.7	0.41	31.1	0.65	32.1	0.90	33.5	0.72
**22.**	**MN324**	57.88	35.0	0.35	33.4	0.45	34.1	0.78	33.6	0.61
**23.**	**MN262**	53.43	34.7	0.20	34.5	0.42	34.1	0.60	34.4	0.50
**24.**	**MN338**	53.43	34.1	0.28	30.3	0.51	32.8	0.77	33.6	0.72
**25.**	**MN345**	57.7	29.8	0.32	30.3	0.51	29.6	0.76	31.1	0.67
**26.**	**MN250**	53.35	31.0	0.29	30.5	0.51	30.6	0.76	31.3	0.70

№	Sample	$T_m(^OC)$	Cp	ECFL	Cp	ECFL	Cp	ECFL	Cp	ECFL
**27.**	**MN377**	53.38	34.6	0.24	32.0	0.49	33.5	0.71	32.7	0.68
**28.**	**MN373**	57.67	35.3	0.18	35.5	0.42	34.1	0.65	35.6	0.52
**29.**	**MN378**	57.79	31.0	0.30	31.6	0.53	30.8	0.80	30.7	0.77
**30.**	**MN226**	53.42	32.5	0.29	32.7	0.51	32.1	0.80	31.9	0.73
**31.**	**MN351**	53.5/57.8	32.4	0.26	30.9	0.52	31.8	0.80	31.3	0.75
**32.**	**MN369**	53.35	32.2	0.34	31.5	0.49	31.5	0.78	31.1	0.56
**33.**	**MN312**	53.36	29.5	0.36	28.8	0.50	28.8	0.72	28.7	0.68
**34.**	**MN329**	53.34	35.1	0.30	33.0	0.44	34.2	0.71	33.5	0.61
**35.**	**MN343**	57.76	33.9	0.27	33.5	0.38	36.3	0.57	30.8	0.17
**36.**	**MN344**	53.5/57.8	30.6	0.29	30.3	0.44	30.4	0.74	30.1	0.62
**37.**	**MN253**	53.42	30.6	0.37	30.1	0.50	30.3	0.79	31.7	0.58
**38.**	**MN255**	57.72	29.8	0.41	29.1	0.52	29.0	0.80	29.0	0.67
**39.**	**MN257**	53.3	31.2	0.28	30.9	0.48	31.3	0.79	31.3	0.66
**40.**	**UZ193**	57.84	30.9	0.21	30.3	0.26	29.9	0.28	29.1	0.28
**41.**	**UZ189**	57.87	33.2	0.20	34.9	0.11	32.1	0.23	32.2	0.38
**42.**	**UZ294**	57.82	30.5	0.27	32.1	0.35	29.8	0.12	29.4	0.48
**43.**	**UZ57**	54.4/57.5	30.1	0.68	31.7	0.73	30.5	0.71	30.6	0.84
**44.**	**UZ111**	57.83	30.8	0.22	32.0	0.34	29.7	0.11	29.5	0.44
**45.**	**UZ177**	53.4/57.9	30.2	0.44	30.5	0.29	30.6	0.38	29.8	0.32
**46.**	**UZ304**	53.4/57.8	28.2	0.66	28.4	0.38	27.7	0.51	27.2	0.48
**47.**	**UZ282**	57.75	30.3	0.24	29.3	0.42	29.0	0.69	28.9	0.54
**48.**	**UZ232**	57.8	34.9	0.23	33.9	0.56	32.9	0.62	32.7	0.47
**49.**	**UZ376**	57.91	33.2	0.48	32.1	0.70	32.0	0.82	30.8	0.64
**50.**	**UZ378**	53.5/57.8	30.3	0.58	31.25	0.87	31.5	0.72	30.8	0.78
**51.**	**UZ379**	53.5/57.8	30.6	0.68	31.2	0.77	30.5	0.74	30.9	0.82
**52.**	**UZ384**	57.9	36.0	0.16	32.6	0.18	31.4	0.27	30.0	0.16
**53.**	**UZ105**	57.8	35.3	0.2	34.6	0.58	34.8	0.4	28.0	0.54
**54.**	**UZ106**	57.6	32.3	0.15	32.2	0.17	32.3	0.38	32.0	0.29

№	Sample	T_m(^{O}C)	Cp	ECFL	Cp	ECFL	Cp	ECFL	Cp	ECFL
**55.**	**UZ107**	53.5/57.9	30.5	0.60	31.8	0.72	30.9	0.75	30.8	0.85
**56.**	**KR008**	53.5	38.4	0.06	36.8	0.20	34.1	0.28	33.4	0.09
**57.**	**UZ232**	57.71	34.9	0.23	33.9	0.56	32.9	0.62	32.7	0.47
**58.**	**KR189**	53.4	32.8	0.28	32.5	0.56	32.3	0.87	31.5	0.72

CP crossing point, ECFL cycle fluorescence
